# A Systematic Review of Youth-to-Parent Aggression: Conceptualization, Typologies, and Instruments

**DOI:** 10.3389/fpsyg.2020.577757

**Published:** 2020-11-30

**Authors:** Izaskun Ibabe

**Affiliations:** Department of Clinical and Health Psychology and Research Methodology, University of the Basque Country UPV/EHU, Donostia-San Sebastián, Spain

**Keywords:** youth-to-parent aggression, child-to-parent aggression, child-to-parent violence, conceptualization, instruments

## Abstract

The goal of this study was to analyze the conceptualization of YPA (youth-to-parent aggression) in relation to terms, definitions, typologies and assessment instruments. To achieve this aim, a systematic review was carried out using the PRISMA protocol. Assessment instruments for YPA were examined in accordance with COSMIN (Consensus-based Standards for the Selection of Health Measurement Instruments). After reviewing the literature on conceptualization and measuring instruments, some gaps were found. The use of some particular terms was justified depending on the age of children and severity of case. Taking into account the theoretical background, a full definition of YPA was offered. Moreover, this study revealed that it was possible to discriminate four typologies of YPA (Offensive, Defensive, Affective, and Situational) as a function of the coercion level and nature of the violence. Eleven instruments to measure YPA were analyzed exhaustively, with the most reported and robust psychometric properties being internal consistency and structural validity, while other validity evidence was understudied. The CPV-Q (12–25 years) obtained the highest rating as a promising instrument. The initial psychodiagnosis of a YPA situation would help in the individual or family intervention, as well as prevent more severe situations of YPA through early intervention.

## Introduction

During last decade, youth-to-parent aggression (YPA) has received growing attention in scientific literature as a result of the progression in complaints filed by parents. This type of family violence puts family safety at risk due to the loss of parental power that it generates, and at the same time the most victimized parents feel guilt and humiliation (Selwyn and Meakings, 2015; Gabriel et al., 2018; Ilabaca and Gaete, 2018). As the number of complaints in Spain of YPA has been stable over the last decade, it is possible that this type of crime has become consolidated as a problem endemic to society (General Prosecutor’s Office of Spain, 2018). In YPA research, it is necessary to operationalize the term “child” because perpetrators older than 18 years are legally considered adults rather than children. In their review of community samples, Simmons et al. (2018) estimated the previous-year incidence of physical YPA between 5 and 21%, usually based on adolescent samples. In the United States, 10% of the assaults committed by young people between 18 and 25 years are against their parents (Snyder and McCurley, 2008). In Spain, 5% of college students perpetrated physical YPA during the past year, taking into account the technical abuse criteria (Ibabe et al., 2020). In a study based on an Australian sample in the 14–25 years age range, 7% of physically abusive behavior toward one parent was reported (Simmons et al., 2019a). All these data reveal the extent of this family and community problem. In order to generalize study results, it is key to specify the age of perpetrators and severity of violent behavior. The consolidation of abusive behavior can gradually lead to the emergence of a criminal trajectory. The Juvenile Court specifies that these offenses are among those presenting the highest problems (General Prosecutor’s Office of Spain, 2018).

One of the best-known definitions of YPA is the one provided by Cottrell (2001). This definition identifies any behavior of a child with the intention of inflicting physical, psychological or financial damage to get power and control over a parent. According Holt (2016), it is an abusive behavior perpetrated toward a parent by a legally recognized child, usually living in the family home. Moreover, Pereira et al. (2017) defined it as a repeated violent behavior, directed toward the parents or the people who act as parents. These definitions show different characteristics, such as intentionality to cause damage, legally recognized child or living at home. The use of different conceptual and operational definitions to study YPA can obscure the true prevalence rates as well as the capacity to identify risk factors for this type of abuse (Simmons et al., 2018). With respect to assessment instruments, Arias-Rivera et al. (2020) analyzed available instruments to measure YPA. Empirical studies with adolescents (10–19 years) in Spanish and English from 2000 to 2017 were examined. Authors identified only two instruments specifically assess YPA, and they concluded it is questionable using measures of interpersonal conflict or violence for the assessment of YPA.

### Objective of the Study

The goal of this study was to provide a systematic review of the conceptualization of YPA (terms, definitions, typologies, and assessment instruments). To achieve this aim, a narrative analysis of papers in the systematic review was carried out with the PRISMA protocol (Urrútia and Bonfill, 2010). To evaluate the quality of the identified instruments, the updated revised COnsensus based Standards for the selection of health Measurement INstruments (COSMIN) methodological guidelines (Mokkink et al., 2018; Prinsen et al., 2018) was applied. Based on the outcomes of the review, this paper discusses the inconsistencies found in conceptualization of YPA, and the best assessment instruments, concluding with suggestions that can advance the understanding of this emergent family violence.

## Methods

**FIGURE 1:**
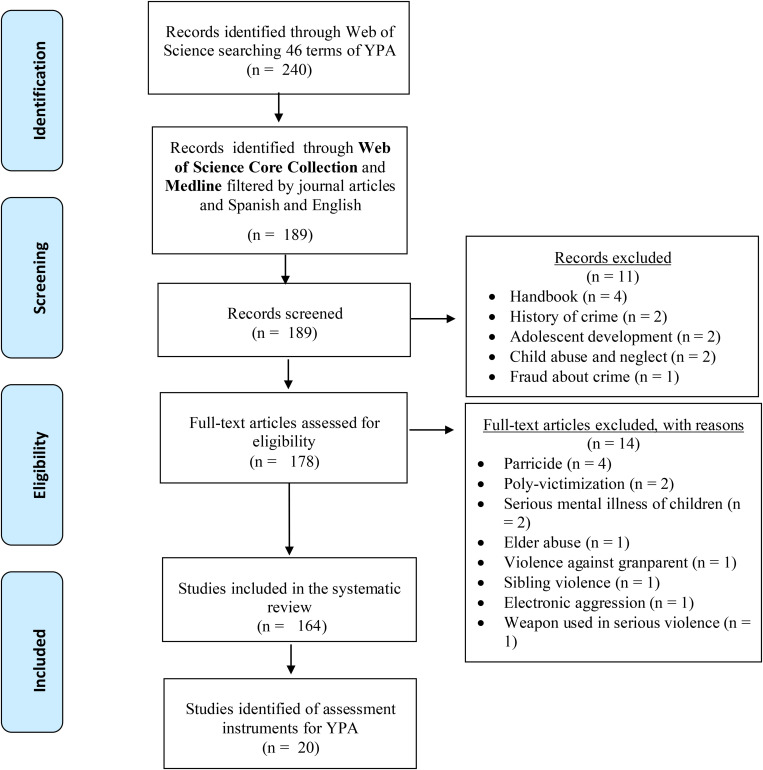
Flowchart of the review process according to Preferred Reporting Items for Systematic reviews and Meta-Analyses (Moher et al., 2009).

This systematic review is based on the PRISMA guidelines with a 27-item checklist. The selection process of the incorporated studies is outlined in the flow diagram (Figure 1).

### Terms, Definitions, Typologies, and Instruments Most Used for YPA

#### Search Strategy

To identify all terms, definitions and instruments potentially pertinent to the review purpose, the searches were conducted in Web of Science (the largest multidisciplinary platform with high-quality studies). On the one hand, Web of Science Core Collection is a select collection of over 21,000 peer-reviewed, high-quality academic journals published worldwide in over 250 disciplines. On the other hand, Medline is the principal database of the U.S. National Library of Medicine, and it includes more than 12 million journal articles in the life sciences. For this reason, the systematic searches were done in the Web of Science Core Collection and Medline (Table 1).

**TABLE 1:** Description of search strategy in Web of Science and results in all databases, without using filters of type of documents or languages.

WEB OF SCIENCE	Results
(TI = (“parent abuse” OR “child-to-parent abuse” OR “child-to-parent violence” OR “child-to-parent aggression” OR “youth-to-parent aggression” OR “youth-to-parent violence” OR “youth-to-parent abuse” OR “youth aggression toward parents” OR “youth violence toward parents” OR “child-to-mother aggression” OR “child-to-father aggression” OR “teenage violence toward parents” OR “adolescent-to-parent violence” OR “adolescent-to-parent aggression” OR “adolescent-parent abuse” OR “adolescent aggression toward parents” OR “adolescent violence toward parents” OR “adolescent abuse toward parents” OR “child-to-father violence” OR “child-to-mother violence” OR “child-initiated family violence” OR “adolescent-initiated parent abuse” OR “battered parent” OR “violence against parents” OR “juvenile domestic violence” OR “adolescent family violence” OR “youth violence in the home” OR “teen violence toward mothers” OR “parents abused by children” OR “adolescent violence in the home” OR “parent-directed aggression” OR “violence by children against mothers” OR “aggression toward mothers” OR “aggression toward fathers” OR “mother abuse” OR “abuse toward mothers” OR “filioparental violence” OR “violence by children toward parents” OR “violence by adolescents toward parents” OR “parents abused by their children” OR “abuse of parents by their adolescent” OR “violence by children against parents” OR “violence by child to parent” OR “violence by adolescent to parent” OR “aggression by child to parent” OR “parents victimized by their children”))	159
OR	
(AB = (“parent abuse” OR “child-to-parent abuse” OR “child-to-parent violence” OR “child-to-parent aggression” OR “youth-to-parent aggression” OR “youth-to-parent violence” OR “youth-to-parent abuse” OR “youth aggression toward parents” OR “youth violence toward parents” OR “child-to-mother aggression” OR “child-to-father aggression” OR “teenage violence toward parents” OR “adolescent-to-parent violence” OR “adolescent-to-parent aggression” OR “adolescent-parent abuse” OR “adolescent aggression toward parents” OR “adolescent violence toward parents” OR “adolescent abuse toward parents” OR “child-to-father violence” OR “child-to-mother violence” OR “child-initiated family violence” OR “adolescent-initiated parent abuse” OR “battered parent” OR “violence against parents” OR “juvenile domestic violence” OR “adolescent family violence” OR “youth violence in the home” OR “teen violence toward mothers” OR “parents abused by children” OR “adolescent violence in the home” OR “parent-directed aggression” OR “violence by children against mothers” OR “aggression toward mothers” OR “aggression toward fathers” OR “mother abuse” OR “abuse toward mothers” OR “filioparental violence” OR “violence by children toward parents” OR “violence by adolescents toward parents” OR “parents abused by their children” OR “abuse of parents by their adolescent” OR “violence by children against parents” OR “violence by child to parent” OR “violence by adolescent to parent” OR “aggression by child to parent” OR “parents victimized by their children”))	194
OR	
(AK = (“parent abuse” OR “child-to-parent abuse” OR “child-to-parent violence” OR “child-to-parent aggression” OR “youth-to-parent aggression” OR “youth-to-parent violence” OR “youth-to-parent abuse” OR “youth aggression toward parents” OR “youth violence toward parents” OR “child-to-mother aggression” OR “child-to-father aggression” OR “teenage violence toward parents” OR “adolescent-to-parent violence” OR “adolescent-to-parent aggression” OR “adolescent-parent abuse” OR “adolescent aggression toward parents” OR “adolescent violence toward parents” OR “adolescent abuse toward parents” OR “child-to-father violence” OR “child-to-mother violence” OR “child-initiated family violence” OR “adolescent-initiated parent abuse” OR “battered parent” OR “violence against parents” OR “juvenile domestic violence” OR “adolescent family violence” OR “youth violence in the home” OR “teen violence toward mothers” OR “parents abused by children” OR “adolescent violence in the home” OR “parent-directed aggression” OR “violence by children against mothers” OR “aggression toward mothers” OR “aggression toward fathers” OR “mother abuse” OR “abuse toward mothers” OR “filioparental violence” OR “violence by children toward parents” OR “violence by adolescents toward parents” OR “parents abused by their children” OR “abuse of parents by their adolescent” OR “violence by children against parents” OR “violence by child to parent” OR “violence by adolescent to parent” OR “aggression by child to parent” OR “parents victimized by their children”))	132
OR	
(KP = (“parent abuse” OR “child-to-parent abuse” OR “child-to-parent violence” OR “child-to-parent aggression” OR “youth-to-parent aggression” OR “youth-to-parent violence” OR “youth-to-parent abuse” OR “youth aggression toward parents” OR “youth violence toward parents” OR “child-to-mother aggression” OR “child-to-father aggression” OR “teenage violence toward parents” OR “adolescent-to-parent violence” OR “adolescent-to-parent aggression” OR “adolescent-parent abuse” OR “adolescent aggression toward parents” OR “adolescent violence toward parents” OR “adolescent abuse toward parents” OR “child-to-father violence” OR “child-to-mother violence” OR “child-initiated family violence” OR “adolescent-initiated parent abuse” OR “battered parent” OR “violence against parents” OR “juvenile domestic violence” OR “adolescent family violence” OR “youth violence in the home” OR “teen violence toward mothers” OR “parents abused by children” OR “adolescent violence in the home” OR “parent-directed aggression” OR “violence by children against mothers” OR “aggression toward mothers” OR “aggression toward fathers” OR “mother abuse” OR “abuse toward mothers” OR “filioparental violence” OR “violence by children toward parents” OR “violence by adolescents toward parents” OR “parents abused by their children” OR “abuse of parents by their adolescent” OR “violence by children against parents” OR “violence by child to parent” OR “violence by adolescent to parent” OR “aggression by child to parent” OR “parents victimized by their children”))	7
**Total documents returned without duplicated publications**	**240**

*TI, title; AB, Abstract; AK, Author keyword (Keywords in their research publications specified by author); KP, Keyword plus (Important terms not listed among the author keywords automatically generated).*

The systematic search was limited to the terms (“parent abuse,” “child-to-parent abuse,” “child-to-parent violence,” “child-to-parent aggression,” “youth-to-parent aggression,” “youth-to-parent violence,” “youth-to-parent abuse,” “youth aggression toward parents,” “youth violence toward parents,” “child-to-mother aggression,” “child-to-father aggression,” “teenage violence toward parents,” “adolescent-to-parent violence,” “adolescent-to-parent aggression,” “adolescent-parent abuse,” “adolescent aggression toward parents,” “adolescent violence toward parents,” “adolescent abuse toward parents,” “child-to-father violence,” “child-to-mother violence,” “child-initiated family violence,” “adolescent-initiated parent abuse,” “battered parent,” “violence against parents,” “juvenile domestic violence,” “adolescent family violence,” “youth violence in the home,” “teen violence toward mothers,” “parents abused by children,” “adolescent violence in the home,” “parent-directed aggression,” “violence by children against mothers,” “aggression toward mothers,” “aggression toward fathers,” “mother abuse,” “abuse toward mothers,” “filioparental violence,” “violence by children toward parents,” “violence by adolescents toward parents,” “parents abused by their children,” “abuse of parents by their adolescent,” “violence by children against parents,” “violence by child to parent,” “violence by adolescent to parent,” “aggression by child to parent,” and “parents victimized by their children”) in topic search (title, abstract, author, keyword, and keyword plus), selecting journal articles published in English or Spanish up to September 2020, 189 journal articles returned.

#### Criteria for Selection

Inclusion criteria were used: (1) academic journals, (2) studies focused on children aged between 10 and 25 years, (3) theoretical and empirical studies, (4) terms in title, abstract or as keywords, (5) studies published in English or Spanish. Exclusion criteria for terms were: (1) parricide studies, (2) not including any research specifically examining elder abuse, and (3) conference proceedings and books.

#### Data Extraction

Retrieved articles from databases were exported to an excel file generated by RefWorks. This file contains information about articles: authors, title, journal, year of publication, abstract, DOI, and link to the article. Titles and abstracts of all the recovered articles were screened. After examining all the references, a list of potential papers was elaborated. These papers were exhaustively evaluated to determine if they satisfied eligibility criteria.

### Identification of Studies Reporting Psychometric Properties

#### Selection Criteria for YPA Assessment Instruments

There were three inclusion criteria for assessment instruments: (1) quantitative measures specifically developed to assess YPA; (2) designed to assess YPA of children aged between 10 and 25 years; and (3) studies published until September of 2020. Meanwhile, exclusion criteria for assessment instruments were: (1) self-report rated by caregivers other than parents; (2) not using instruments to assess YPA within judicial samples (e.g., juvenile court records of YPA); (3) qualitative methods used to assess YPA; (4) instruments without information about psychometric properties.

#### Data Extraction

All papers that fulfilled eligibility criteria for systematic review were analyzed again to select papers fulfilling criteria for assessment instruments. For this selection, the Method section of each paper was examined.

#### Evaluation of the Quality of Informed Psychometric Properties

COSMIN guidelines (Mokkink et al., 2018; Prinsen et al., 2018) was applied to evaluate the quality of the selected instruments. This checklist is composed of ten psychometric sections (e.g., structural validity, criterion validity, internal consistency, reliability, cross-cultural validity, among others). Finally, instruments were classified according to global quality of evidence and results: Category A (recommended), Category B (may be used with caution) and Category C (not recommended).

## Terms Used for YPA

There is marked variability in the way YPA is referred to in the scientific literature from 1957 until 2020 (Table 2). Terms such as parent abuse, parental aggression, or parental violence have been used to indicate YPA (Cottrell, 2001; Murphy-Edwards, 2016), but these terms can be confused with child abuse by parents. Child-to-parent violence has been popularized in the recent scientific literature. However, due to types of behaviors that its definition includes (psychological, emotional, or financial abuse), the term should be designated as aggression or abuse rather than violence. Results of the search indicate that the most used terms are: child-to-parent violence and parent abuse. However, the use of child-to-parent violence does not seem adequate because physical and psychological aggressions are integrated in this context. Violence is an act of physical force that causes or is intended to cause harm, whereas aggression is a hostile behavior that may be physical, verbal, or passive. Abuse is defined as any action involving physical violence or emotional cruelty that intentionally harms or injures another person. In abusive behavior there is usually an abuser and a victim, but there are no clear cut-off points to consider a child abusive rather than just aggressive (Gallagher, 2008).

**TABLE 2:** Descriptors in the selected published papers and search results in Web of Science and Google Scholar.

Number	Descriptors/levels	Web of science in topic	Google scholar
1.	“Child-to-parent violence”	99	1,080
2.	“Parent abuse”	69	2,320
3.	“Violence against parents”	21	633
4.	“Child-to-parent aggression”	19	256
5.	“Adolescent-to-parent violence”	17	458
6.	“Mother abuse”	12	1,090
7.	“Child-to-mother violence”	11	225
8.	“Child-to-parent abuse”	9	149
9.	“Adolescent family violence”	8	116
10.	“Adolescent violence in the home”	6	170
11.	“Parent-directed aggression”	6	57
12.	“Adolescent violence toward parents”	5	411
13.	“Battered parent”	5	383
14.	”Child-to-father violence”	5	25
15.	“Aggression toward mothers”	4	302
16.	“Violence by children against mothers”	1	266
17.	“Aggression toward fathers”	1	158
18.	“Abuse toward mothers”	1	114
19.	“Child-initiated family violence”	1	108
20.	“Adolescent-initiated parent abuse”	1	90
21.	“Teenage violence toward parents”	1	82
22.	“Youth violence toward parents”	1	53
23.	“Parents abused by their children”	1	32
24.	“Youth-to-parent aggression”	1	28
25.	“Youth violence in the home”	1	28
26.	“Filioparental violence”	1	23
27.	“Child-to-mother aggression”	1	9
28.	“Adolescent-parent abuse”	1	7
29.	“Child-to-father aggression”	1	4
30.	“Juvenile domestic violence”	0	101
31.	“Adolescent aggression toward parents”	0	59
32.	“Parents victimized by their children”	0	36
33.	“Violence by children against parents”	0	30
34.	“Adolescent-to-parent aggression”	0	27
35.	“Youth-to-parent violence”	0	24
36.	“Abuse of parents by their adolescent”	0	23
37.	“Youth-to-parent abuse”	0	12
38.	“Parents abused by children”	0	8
39.	“Youth aggression toward parents”	0	5
40.	“Adolescent abuse toward parents”	0	4
41.	“Violence by children toward parents”	0	4
42.	“Violence by adolescents toward parents”	0	4
43.	“Teen violence toward mothers”	0	2
44.	“Violence by child to parent”	0	1
45.	“Violence by adolescent to parent”	0	1
46.	“Aggression by child to parent”	0	1

The term child-initiated family violence (Peek et al., 1985) and adolescent-initiated parent abuse (Hong et al., 2012) are singular because they point out that the child initiates the abuse toward parents. Although child-to-parent abuse is frequently used in the scientific literature, when the perpetrators of this type of violence are young adults, it is not an appropriate term. In this review, the proposed term to use in the future is *youth-to-parent aggression* because adolescents and young adults are included, and the term aggression integrates minor aggression and severe maltreatment. Additionally, it would not be appropriate to generalize the findings of early childhood aggression toward parents to older children’s aggression because of differences in the developmental period and parenting (Simmons et al., 2018), as well as in the legal consequences for children and parents, or the harm caused. Thus, it could become a new line of research, using the term *child-to-parent aggression* when the children are younger 12 years to investigate early aggressive behavior of children. Moreover, it would be interesting to study the aggressive behavior of adult children toward their parents.

## Conceptualization of Youth-To-Parent Aggression

The inconsistency of the YPA definitions is one of the major gaps in developing scientific knowledge (Simmons et al., 2018). The first definitions of YPA appear in the scientific literature referring to the battered parent syndrome to illustrate the effects of parent abuse by children (Sears et al., 1957; Harbin and Madden, 1979). According to Bobic (2002), most definitions of YPA are derived from domestic violence terminology due to the similarities in the power issues and the tactics used. In Table 3, different definitions of YPA and their characteristics are shown. Harbin and Madden (1979) defined youth-to-parent violence as a type of family violence perpetrated by adolescents and young adults. However, in other definitions, the terms child under age 18 (Calvete et al., 2015a), teenage child (Cottrell, 2001), or adolescent child (Cottrell and Monk, 2004) are specified, but in other definitions, the perpetrator’s age or his o her development stage is not mentioned (Paterson et al., 2002; Aroca-Montolío et al., 2014; Pereira et al., 2017). Variations in children’s age to define the target population could limit the generalizations of the extent of this family abuse. There is little research which includes perpetrators over 18 years, legally considered adults (Edenborough et al., 2011; Gámez-Guadix and Calvete, 2012; Simmons et al., 2019a, b; Ibabe et al., 2020), even though at least a half of the children in the 18–24 years age range continue living with their parents according to data of different countries (Simmons et al., 2018). The cohabitation between perpetrator and target should be an inclusion criterion in the YPA definition more relevant than applying an arbitrary age-based criterion. Unfortunately, YPA does not disappear when children reach adulthood, and legal consequences for adult perpetrators of YPA could be more serious than for child perpetrators. In any case, it would be interesting to research adult children’s abuse toward their parents.

**TABLE 3:** Definitions of YPA and their characteristics.

Studies	Definitions	Characteristics
Aroca-Montolío et al., 2014	Intentional and conscious behavior of children with the desire to cause harm, prejudice, or suffering to their parents, repeatedly, and with the immediate aim of gaining power, control, and domination over their parents to get what they want through psychological, economic, or physical violence	Repeated behavior Intentionally Consciously Power and control Economic violence
Brule, 2007	Repetitive verbal, physical, and emotional harm inflicted by 11 to 17-year-old adolescents toward parent/s legally and socially responsible for their abuser	Repetitive behavior Adolescents
Calvete et al., 2015a	Behavior perpetrated by a child under age 18 intended to cause physical, psychological, or financial harm to their parent or guardian	Child under age 18 Financial harm Guardians as victims
Clarke et al., 2017	A persistent pattern of abuse that enables young people to assert power and control over their parents	Persistent pattern of behavior Abuse Young people
Cottrell and Monk, 2004	Any action by adolescents aimed at causing economic, psychological, or physical harm to parents and/or persons occupying their place	Adolescents Economic harm
Cottrell, 2001	Any harmful act (physical, psychological, or financial) by a teenage child that is intended to gain power and control over a parent	Teenage Financial harm Power and control Intentionally
Harbin and Madden, 1979	It is a subtype of family violence with both physical assault and serious threats of physical harm by children and young people	Children and young people
Holt, 2011	Physical, psychological or financial damage caused by an older child to a parent with the intention of controlling the relationship	Older child Controlling the relationship
Holt, 2016	Abusive behavior perpetrated toward a parent by a son or daughter who is legally recognized as a child, and who is usually still living in the family home	Child or legally recognized as a child Living in the family home
Howard and Rottem, 2008	Adolescent violence toward parents takes diverse forms: physical violence, destruction of property and/or possessions, threats and intimidation, psychological, emotional and social abuse, financial abuse and sometimes sexual abuse	Destruction of property and/or possessions Financial abuse Sexual abuse
Miles and Condry, 2015	It is a continuum of behavior ranging from teenagers verbally abusing and using threats of violence toward their parents to damaging parental property and physically assaulting them	Continuum verbal abuse-threats - property damage - physical assault Teenager
Paterson et al., 2002	Any act perpetrated by a child that makes their father/mother feel threatened, intimidated, and controlled	Parents feel threatened and controlled
Pereira et al., 2017	Repeated behavior of physical, psychological, or economic aggression, directed toward the parents or the people who occupy their place, excluding aggressions with a state of diminished consciousness	Repeated behavior Intentionally Consciously Economic violence Guardians as victims

Intentionality or the consciousness of harm to parents should be a condition for considering youth-parent aggression, as some authors have suggested (Cottrell, 2001; Aroca-Montolío et al., 2014; Pereira et al., 2017). Thus, those cases in which there is a transitory or permanent lack of conscience (general sense of right and wrong and feeling of guilt because the person knows they have done something wrong) should be excluded. The state of diminished consciousness can be due to serious mental illness, substance intoxication or mental deficiency. Moreover, another condition for YPA to be considered is that the episodes of aggressive behavior toward parents are repeated, as specified in two definitions (Aroca-Montolío et al., 2014; Pereira et al., 2017). Thus, isolated aggressive behavior by children should be excluded. In normal development, adolescents make every effort to individuate from their parents, and young people could become defiant (Kennair and Mellor, 2007), but defiance does not imply abuse. Some authors consider that there is YPA when children attempt to achieve control and power over a parent (Cottrell, 2001; Paterson et al., 2002; Aroca-Montolío et al., 2014), but parricide (killing one’s parents) should be excluded. Abusive behavior is coercive and is perpetrated against someone less powerful (Gallagher, 2004). Can children abuse their parents, when objectively parents have far more power? According to Gallagher (2004), children are abusive when their behavioral pattern is aimed at controlling or disempowering the parent. Nevertheless, all children who batter or even injure a parent are violent, but are not necessarily abusive (e.g., self-defense, outbursts of anger, aggression by a severely disabled child, or aggression by drug-affected or psychiatrically disturbed children). Therefore, the characteristic of power and control will not be an essential condition for YPA, but this idea will be developed in the YPA typologies section.

Some definitions point out that the victims can be the parents or those who exercise their function (Calvete et al., 2015a; Pereira et al., 2017). Moreover, the perpetrator of YPA could be a biological child or an adopted child (Holt, 2016). The characteristic of cohabitation was also mentioned (child perpetrator typically still residing in the family home) (Holt, 2016). Concerning the nature of behaviors that are thought to compose YPA, there is considerable variability in the severity of assaults or damage caused, ranging from verbal aggression (e.g., yelling at parents) to severe physical aggression (e.g., using a knife on parents), and for such assaults, a child can be incarcerated. The full range of aggression (physical, emotional, psychological, and financial) is included in YPA. These categories could overlap. Physical violence is not conceived without emotional violence, given the fear or perception of helplessness on the part of the victim. Financial aggression has been mentioned in most definitions (Cottrell, 2001; Aroca-Montolío et al., 2014; Calvete et al., 2015a; Pereira et al., 2017), and has sometimes been included as a component of psychological aggression (Calvete et al., 2013a), but on other occasions it is assessed as a concept in itself (Ibabe, 2014; Ibabe et al., 2014) or as a part of coercive behavior (Simmons et al., 2019b). Child-to-parent sexual aggression has been taken into account in two studies (Howard and Rottem, 2008; del Moral et al., 2015). Some social services professionals highlight the existence of sexual violence as a kind of YPA (del Moral et al., 2015). In official complaints, the sexual abuse perpetrated by the children may be concealed by their parents, perhaps due to feelings of shame and guilt, and to safeguard the image of the family itself. However, taking into account that, in the definition of interpersonal violence, sexual harm is mentioned specifically (World Health Organization, 2020), it is questionable that sexual violence does not appear in YPA definitions. Perhaps sexual YPA is unlikely, but has this type of violence been analyzed? Therefore, this type of violence should not be ruled out because of a lack of empirical evidence in previous studies of youth who perpetrated YPA. It is important to take into account that sexual abuse by children was found in children who perpetrate YPA (Sheehan, 1997; Cottrell and Monk, 2004).

To conclude this section, we underline that the definition of YPA should include eight characteristics: (a) repeated aggression, (b) consciously, (c) intentionality, (d) the perpetrator is a youth, (e) the victim is a parent or caregiver, (f) the child is biological or adopted, (g) the child usually lives in the family home, and (h) physical and non-physical aggression. The full definition proposed in this study for YPA is: Young people/children who consciously direct physical, psychological, emotional, financial, or sexual aggression toward one parent or caregiver, repeatedly over time, when the perpetrator and the victim habitually live together. Consequently, the following cases should be excluded from this definition: children younger than 12 years, isolated incidents of child-to-parent aggression, when the children do not habitually live in the family home, when there is no consciousness of the damage caused to their parents (severely disabled children, psychiatrically disturbed children, or drug dependence), parricide, aggressions toward siblings, grandparents, or other members of the extended family. Aroca-Montolío et al. (2014) indicated that the immediate aim of YPA is to gain power, control, and dominance over the parents, and Paterson et al. (2002) defined YPA as any act perpetrated by children that makes their parents feel threatened, intimidated, and controlled. If YPA is constructed as an abuse of power, developing appropriate intervention strategies to empower parents to restore control over their situations will be required. Nevertheless, the characteristics of power, control, and dominance have not been added to the full definition. As explained below, there are different typologies of YPA, and this characteristic is not present in all.

## Typologies of YPA

Traditionally, instruments that assess the perpetration of violent behavior have been criticized because they do not take into account the context or reasons that motivate this behavior (Calvete et al., 2007). Some studies analyzed the most frequent reasons for YPA attacks (Calvete and Orue, 2016; Contreras et al., 2019). Calvete and Orue (2016) found that the most frequent reasons for YPA in Spanish adolescents between 14 and 18 years are divided into three groups: instrumental motives (to obtain a benefit by the adolescent), affective motives (emotional experience of anger and other experiences such as feeling misunderstood by parents), and defensive motives (self-defense and defending other people). However, in a Spanish sample with adolescents (12–18 years), Contreras et al. (2019) found two factors related to the reasons for YPA: instrumental and reactive aggression.

In intimate partner violence situation, aggression that partner violent men perpetrate can be explained by the need to control the partner or by emotional reactivity (Ross and Babcock, 2009). Johnson (2008, 2011) established four typologies as a function of the coercive control, severity, frequency, and physical harm of the assaults (intimate terrorism, violent resistance, mutual violent control, and situational violence). YPA abusers constitute a heterogeneous group (Contreras and Cano, 2014), and taking into account the above-mentioned points of view, four typologies of YPA can be distinguished depending on the coercion level and directionality of the violence, with the child as perpetrator: offensive (abusive/instrumental), defensive, affective, and situational (conflictive parent-children relationship). In the proposed typologies, there are specific psychological perpetrator profiles and intervention needs. Moreover, it would be interesting to recognize the dyadic nature of YPA with two mutually exclusive categories: unidirectional youth-to-parent (a youth is a perpetrator and a parent is a victim) and bidirectional (a youth is a perpetrator and a victim at the same time).

### Offensive YPA

This typology of YPA includes unidirectional child-to-parent abuse and is similar to the intimate terrorism described by Johnson (2008) as a systematic and controlling abuse pattern of male-perpetrated gender violence. In this case, youth/children exercise coercive control or emotional violence toward their parents. Parents in a YPA situation live under a constant threat. In a qualitative study on parents who are experiencing YPA, one mother briefly described the situation: “my son is now the terrorist in my home” (Holt, 2013). Children have the intention to obtain power and control over a parent, and most of the definitions of YPA mention this characteristic (Cottrell, 2001; Paterson et al., 2002). This is not a spontaneous child behavior but instead implies premeditation and manipulation by the child. This type of YPA is characterized by proactive aggression, also called instrumental aggression, which means the perpetrator’s behavior is planned, predatory, and cold-blooded (Ramírez and Andreu, 2006) and is perpetrated in the absence of anger (Merk et al., 2005). This point of view is consistent with the instrumentality that has been often described by professionals involved with youth performing aggressive behavior toward their parents (Howard et al., 2010). In these cases, interventions should help the affected families to empower parents to control their children’s behavior.

Some authors indicate that the instrumental role of YPA is related to permissive parenting and lack of limits for children as well as to the culture of consumption in current Western societies (Calvete et al., 2013b). “Youth entitlement” is consistent with proactive aggression (Howard et al., 2010), because young people feel it is their right to exert controlling and aggressive behavior to gain whatever they desire. In this context, YPA may represent a way to get aims when the parents decline to carry on satisfying the children’s desires. This typology has some similarities with intimate terrorism concerning abusive power and control over the victim, but the main difference is the power balance: equal power for intimate partner relationships and unequal power for YPA. Generally, people with Antisocial Personality Disorder (ASPD) have positive views of violence and tend to consider their couples as objects to be controlled (Ross and Babcock, 2009). They are also described by their manipulation of others for personal achievement, as well as by their constant disrespect and abuse to others (American Psychiatric Association, 2000). An antisocial profile in children has also been found in YPA studies (Ibabe, 2014; Ibabe et al., 2014).

Knowledge about intimate terrorism applied to YPA indicates that the cases reported by the parents or families who ask for help in mental health services for this problem are the more serious cases. Moreover, gender asymmetry of the perpetrator and the victim appears in these cases, sons are the most frequent perpetrators while mothers are the most frequent victims. In addition, children may use strategies based on control and manipulation to gain power over their parents. In the first definitions of YPA, the goal to achieve power and control over parents was present. In this situation, parents would lose authority and would worry about their safety and that of their family.

### Defensive YPA

When aggression is a direct answer to an assault or is mainly proposed to avoid another assault toward oneself or another, it is considered defensive. This type of violence is bidirectional because the young person involved has direct or indirect experience of victimization, and it is related to violent resistance. Defensive YPA would include violent behavior for self-defense if the child has experienced parent-to-child abuse (including aggressive discipline and neglect) or for defending another person in interparental violence situations. Some adolescents or young adults who perpetrate YPA were abused or neglected by their parents, and, in particular, they experienced their father’s application of physical punishment (Browne and Hamilton, 1998; Calvete et al., 2015b; Ibabe and Bentler, 2016; Ibabe, 2019) or were exposed to interparental violence (McCloskey and Lichter, 2003; Boxer et al., 2009; Ibabe and Jaureguizar, 2011; Gallego et al., 2019; Ibabe et al., 2020). Some young people intervene to avoid intimate partner abuse against their mothers (Gallagher, 2008). When children witness gender violence, they may defend their mother and direct their aggression toward their father. Browne and Hamilton (1998) found that 80% of physical YPA happened in the context of child abuse. All of these results strongly suggest a reciprocal relationship between child abuse and YPA, however it is necessary to get evidence about reciprocal effects (immediate or close in time) that may also explain this relationship (Gallego et al., 2019). Family violence exposure can have an effect on YPA through social information processing (Simmons et al., 2018). For example, experiencing violence was associated with more negative perceptions and expectations of social relationships (Contreras and Cano, 2016). In defensive YPA situations, family intervention would have to include intervention with parents to reduce aggressive discipline or neglectful practices.

### Affective YPA

Affective aggression is described as being impulsive, spontaneous, hostile, affective, and hot-blooded (Ramírez and Andreu, 2006), and occurs in reaction to a supposed threat and in the presence of intense rage (Dodge and Coie, 1987). Children were often described by their parents as having “anger issues” and not being able to cope and or control themselves (Holt, 2013). There is even an inclination to think that most YPA is affective (expressive) rather than controlling, particularly when the children have suffered trauma in early childhood (Gallagher, 2008). Some psychological disorders, psychological distress, or substance use of young people may be the cause of conflicts between parents and children. This typology of YPA is unidirectional violence; at least, there is no parent-to-child abuse or interparental abuse. Although the parents may use violence to defend themselves, the authorities (childhood and family services, domestic violence, police, and courts) may rigorously penalize any such defensive violence by the parents and unconsciously absolve the aggressive children in morally ambiguous situations (Gallagher, 2008).

Concerning mental health, Borderline Personality Disorder is distinguished by emotion dysregulation, profound fear of abandonment, difficulty controlling anger, and unstable interpersonal relationships (American Psychiatric Association, 2000). Perpetrators with this disorder may perpetrate physical violence against their parents when they become distressed as a way of regulating negative feelings, similar to intimate partner abuse situations (Keltner and Kring, 1998). Problem drug use frequently produces negative effects both for drug users themselves and for their family members (Orford et al., 2013). Drug use and YPA are positively associated according to a vast majority of studies in a clinical context (Routt and Anderson, 2011; Contreras and Cano, 2014; Ibabe et al., 2014) and community population (Simmons et al., 2018). Drug problems may be associated with an antisocial profile (Ibabe, 2014; Simmons et al., 2018), including property damage in the parent’s home or personal belongings of parents (Margolin and Baucom, 2014) or financial abuse (Ibabe et al., 2014). In affective YPA, interventions centered on anger management and development of social skills in young people (Brown and Parsons, 1998), treatment of substance use problems or dependence, as well as training of parents in strategies of positive communication with their children would be recommended (Calvete and Orue, 2016).

### Situational YPA

Situational violence could occur in parent-child relationships, although this has not been studied empirically. This type of violence is of low intensity, and often the consequence of a situational conflict rather than a tool for controlling or self-defense. It involves a minor form of bidirectional violence without the abuse of power by parents or children, but with conflictive parent-child relationships. Situational YPA is due to the inability to cope in conflictive situations. Some conflictive context turns into an argument that turns into verbal aggression and, eventually, physical violence. This means that both parents and children keep losing their control during an argument, and this may lead to increased occurrences of violence. Parents and children may be unskilled at arguing, listening to each other, or are not sufficiently socially skilled, and lose control over themselves. If they are frequently confronted with this type of violence, a feeling of inability to cope with these specific situations may develop.

Although this typology of YPA is different from abusive YPA, it still has great potential to hurt family members and their relationships. This type of violence could be the most common form of YPA. When this type of pattern occurs, arguments escalate to minor violence. Disputes can progress to yelling or insults, then to actions like throwing belongings or pushing each other (Johnson and Leone, 2005). Families experiencing situational violence can be helped by early intervention for YPA situations (Ibabe et al., 2018) carried out by trained mental health professionals, in which they learn effective conflict-resolution and communication skills strategies. It is important to indicate that in a small number of families there is serious reciprocal abuse, where adolescents may have fights with their fathers but be abusive and controlling toward their mothers (Gallagher, 2008).

## Assessment of YPA

It is essential to obtain an instrument to measure a varied range of YPA behaviors, integrating all of the elements included in the conceptualization. Below, the most frequently used instruments to assess YPA are described with psychometric studies when available (Table 4).

**TABLE 4:** Instruments to assess YPA with available psychometric studies.

Instrument/study	Type of sample, sample size, age, and country	Dimensions	Number of items/Reporting period	Psychometric properties	TSR/Cat
1. Violent behavior questionnaire (Paterson et al., 2002)	Clinical population Intervention group for mothers (*n* = 14) Australia	Physical verbal Socio-emotional life threats	22 descriptors	Face validity for each item	?C
2. Adolescents’ parent-directed aggression (Margolin and Baucom, 2014)	Community population 112 parents with a child aged 9–10 years California	Physical aggression property damage verbal aggression	14 items	α = 0.54–0.75	?C
3. CTS for YPA (Calvete et al., 2011)	Community sample: 1,427 12–17 years Spain	Physical Verbal	6 parallel items Previous 12 months	α = 0.66 α = 0.88	++B
(Gámez-Guadix and Calvete, 2012)	University students: 1,861 participants Spain	Physical Psychological	6 parallel items Previous 12 months	α = 0.74 α = 0.79	
(Lyons et al., 2015)	University students: 365 participants Canada	Child-to-mother verbal Child-to-father verbal	6 items When children were 10 years old	α = 0.64 α = 0.65	
(Beckmann, 2020)	3,548 adolescents Germany 9th grade students	Physical Psychological	4 parallel items Previous 12 months	α = 0.67 α = 0.76 EFA One factor	
4. APQ (Ghanizadeh and Jafari, 2010)	Clinical sample: 74 children 5–14 years Iran	Physical-financial Psychological Verbal	27 items Previous 2 months	EFA KMO = 0.75 Varimax Three-factor solution: 51.8% variance α = 0.78–0.93	+++B
(Fawzi et al., 2013)	Clinical sample: 150 children 13–19 years Egypt	“	“	Concurrent validity *r* = 0.85 α = 0.77–0.90	
5. IVS (Ibabe and Jaureguizar, 2011)	Community sample: 485 adolescents 12–18 years Spain	Physical Psychological Emotional	9 items Previous 12 months	EFA Three-factor solution, 63% variance CFA: First (intra-family violence) and Second order latent factor (physical, psychological and emotional), CFI = 0.95, IFI = 0.95, NNFI = 0.94, RMSEA = 0.054 Overall α = 0.80	+++B
(Ibabe et al., 2014)	Clinical sample: 106 adolescents Community sample: 125 adolescents 14–18 years Spain	Physical Psychological Emotional Financial ^a^	7 items Previous 12 months	Principal Component Analysis, 88% variance, Three factor solution α = 0.85–0.88	
6. CVS (Edenborough et al., 2011)	Community sample 10–24 years Pilot study: 129 mothers Study: 1,024 mothers Australia	Child-to-mother violence	24 items Previous 12 months	EFA: ML Unidimensional α:0.98–0.99 Test-retest reliability ICC:0.97	+++B
7. Risk assessment (CPVR) (Loinaz and Sousa, 2020)	Clinical (60) and judicial (31) contexts Spain 91 participants 13–28 years	Type of violence Psychological profile of the aggressor Social adaptation of the aggressor Family factors	24 risk factors 6 protective factors	Test-retest > 0.90 Inter-rater > 0.90 Judicial and clinical contexts (AUC = 0.83) Injuries to the mother (AUC = 0.76) 69% high risk -judicial context- 81% low risk -clinical context-	+++B
8. Parent abuse scale (Girl-mother version) (Abbaspour et al., 2019)	Community population 188 high school’s mothers Iran	Emotional abuse Physical abuse	15 items	EFA KMO = 0.89 Two factors CFA: two-factor solution: CFI = 0.97, GFI = 0.91, RMSEA = 0.07 α = 0.75–0.93	+++B
9. CPAQ (Calvete et al., 2013a)	Community sample: 2,719 adolescents 13–18 years Spain	Physical Psychological	10 parallel items Previous 12 months	CFA CFA: Two-factor solution, CFI = 0.99, RMSEA = 0.048 α = 0.73–0.76	++++B
(Calvete et al., 2017)	Community sample: 880 adolescents and 880 parents 13–19 years Spain	Physical adolescents Physical parents Psychological adolescents Psychological parents	10 parallel items –adolescents- 10 items –parents- Previous 12 months	CFA Four-factor solution: CFI = 0.99, RMSEA = 0.06 α = 0.55–0.83 adolescents α = 0.56–0.86 parents	
(Del Hoyo-Bilbao et al., 2018)	Clinical sample 169 12–24 year old Spain	Physical v father Physical v mother Psychological father Psychological mother	10 parallel items Previous 12 months	CFA Four-factor solution: NNFI = 0.981, CFI = 0.985, RMSEA = 0.068 α = 0.79–0.84	
(Calvete and Veytia, 2017)	1,417 adolescents 14–19 years old México	Physical v father Physical v mother Psychological father Psychological mother	10 parallel items Previous 12 months	CFA Four-factor solution: NNFI = 0.989, CFI = 0.991, RMSEA = 0.067 α = 0.83–0.89	
10. ABC-I (Simmons et al., 2019a)	Community sample 14–25 years Study 1: 374 parents Study 2: 587 children Australia	Verbal aggression Physical aggression Coercive behavior	9 parallel items Previous 12 months Score ≥ 16 abusive	Principal Component Analysis KMO = 0.78; three-factor solution, 72% variance Criterion validity: parents’ judgments (*r* = 0.22–0.53) PLS-SEM Convergent validity: mothers (ρ = 0.47) and fathers (ρ = 0.51) ROC analysis: Sensitivity = 0.82; Specificity = 0.83	++++B
11. CPV-Q (Contreras et al., 2019)	Community sample: 1,386 adolescents 12–18 years Spain	Psychological Physical Financial Control/domain	14 parallel items Previous 12 months	EFA KMO = 0.88 Four-factor solution: 41% variance CFA: Four-factor solution, CFI = 0.97, TLI = 0.96, RMSEA = 0.04–0.05 α = 0.70–0.88	+++++B
(Jiménez-García et al., 2020)	823 university students 18–25 years Chile	Psychological Physical Financial Control/domain	19 parallel items Period 12–17 years	CFA Four-factor solution (mothers/fathers): CFI = 0.94–0.96, TLI = 0.93–0.95, RMSEA = 0.02–0.04 α = 0.71–0.83 Convergent validity with support and affection	

*^*a*^An item was added to the original scale for assessing “financial violence” (“I steal money or things from my parents”); EFA, Exploratory Factor Analysis; CFA, Confirmatory Factor Analysis; +, sufficient; ?, indeterminate; TSR, Total sufficient rating; Cat., Categories for recommendations on suitable instruments (Prinsen et al., 2018); B, Instrument in need of further validation, may be used with precaution (Prinsen et al., 2018).*

### Conflict Tactics Scale (CTS; Straus, 1979; CTS-PC; Straus et al., 1998)

This scale is the most widely utilized instrument to measure aggressive behavior among all family members. The CTS is designed to get data on all possible dyads among family members, and it measures physical aggression, psychological aggression and injury during the previous year. It has been adapted to measure physical and verbal aggression against parents (Calvete et al., 2011; Gámez-Guadix and Calvete, 2012; Beckmann et al., 2017). Gámez-Guadix and Calvete (2012) applied the CTSCP with 6 items proposed for the International Parenting Study to assess YPA. These items are originally from the CTSPC (Conflict Tactics Scales—Parent-Child) (Straus et al., 1998), in which the goal was to improve the scales to measure parent-child conflicts. Three items indicate verbal aggression (cursing, yelling, and threatening to beat up the parents), while the other three indicate physical aggression (slapping, kicking, and hitting with an object that may cause damage), in relation to the last 6 months, using a scale from 0 (*Never*) to 2 (*Often*). Although in original studies this scale was administrated to children from 3 to 25 years of community sample, in some studies it has also been applied to graduate students (18–25 years) (Gámez-Guadix and Calvete, 2012).

### Abused Parent Questionnaire (APQ; Ghanizadeh and Jafari, 2010)

This instrument measures four types of abuse: physical (e.g., your child’s hitting you), psychological, verbal, and financial abuse. The parents and their children give information concerning the frequency of the executed behaviors by children during family conflicts in the preceding 2 months. The response categories ranged from 0 (*Never*) to 6 (*More than 20 times*). Three types of abuse (physical-financial, psychological, and verbal) were found in an exploratory factor analysis. This scale was administrated to children of 3–25 years from clinical population.

### Intra-Family Violence Scale (IVS; Ibabe and Jaureguizar, 2011; Ibabe et al., 2014)

This instrument includes a child-to-parent abuse subscale that measures physical (“During quarrels with my father/mother, I have pushed or hit him/her”), psychological (“I insult or threaten my father/mother when I get angry for any reason”), and emotional abuse (“I blackmail my father to get what I want”) toward parents with 3 parallel items (father/mother) with a 5-point Likert scale (1 = *Never*, 5 = *Many times*). The three-factor structure was obtained by exploratory and confirmatory factor analysis (Ibabe and Jaureguizar, 2011; Ibabe et al., 2014). Moreover, the difference between psychological and emotional abuse is theoretically supported by some studies (Cottrell, 2001; Kennair and Mellor, 2007; Howard and Rottem, 2008). The internal consistency of the three subscales was adequate (α > 0.70). The subscale has an item to measure financial abuse (“I steal money or things from my parents”). This scale was administrated to adolescents of 12–18 years from community and clinical population.

### Child-to-Mother Violence Scale (CVS; Edenborough et al., 2011)

This scale explores respondents’ experiences of child-to-mother violence with 12 items (e.g., Making her [the mother] think she was crazy), and with four response options for each item (*Never*, *Occasionally, Most weeks*, and *Daily*). There are additional questions about the mother’s actions following the abuse, and support networks. A maximum likelihood factor analysis supported a single underlying construct. This scale was administrated to children of 10–24 years from a community population.

### Child-to-Parent Violence Risk Assessment (CPVR; Loinaz and Sousa, 2020)

This risk assessment tool was elaborated according to international quality standards (Douglas et al., 2014). The instrument is comprised of 24 risk factors categorized into four dimensions (type of violence, psychological characteristics of the perpetrator, adaptation of the perpetrator, and family factors), and six protective factors. Each risk factor can be present, partially present, or absent for the present time (during the last year) and for the past. Furthermore, this instrument contains more than 20 possible risk factors (i.e., single-parent family, adoption, academic situation, immigration, parent’s criminal histories, and so on). The best results in prediction of low and high risk was for injuries to mother with a cut-off score situated between 22 and 23.

### Adolescent Child-to-Parent Aggression Questionnaire (CPAQ; Calvete et al., 2013a)

This instrument has 10 parallel items (father/mother) to assess psychological (7 items; e.g., “You have blackmailed your mother/father to get what you wanted”) and physical aggression (3 items; e.g., “You have pushed or hit your mother/father in a fight”) during the past year. The answer format was based on a 4-point Likert scale (0 = *Never*, 3 = *Six or more times*). Severe physical aggression is considered if physical aggression has occurred at least *three times in the last year*, while severe psychological aggression is considered if psychological aggression has occurred at least *six times in the past year*. This instrument also consists of a measurement of the reasons for the aggression (e.g., “If you indicated that you hit your father or your mother in one of the preceding questions, please state the reasons for this”). The authors specified that this instrument could be useful as a screening tool to evaluate the presence of YPA or as a measure to study effectiveness of an intervention. This scale was administrated to children of 13–18 years from a community population.

### Abusive Behavior by Children-Indices (ABC-I; Simmons et al., 2019a)

This instrument was created to differentiate normative behavior toward parents from YPA, taking into account the frequency and severity of the behavior. It has 9 behavior descriptors rated by frequency on a 6-point Likert-type scale (1 = *Never*, 6 = *Daily*) over 12 months with three factors: Physical Aggression (3 items), Verbal Aggression (2 items), and Coercive Behavior (4 items; e.g., “Stole money or possessions from parents,” “Threatened to hurt myself or others if the parent did not do what the child wanted”). Participants who get 16 scores or greater are categorized as abusive. The ABC-I scoring system differs by item, based on parents’ perceptions to be considered abusive depending on the frequency (e.g., “Shouted or swore at a parent”; Daily = 16 scores) (Instructions for administering see Simmons et al., 2019b). This instrument can be used with adolescents and young adults aged 14–25 years and their parents but should be administered together with the BACPAQ (Beliefs About Child-to-Parent Abuse Questionnaire) to assess perceptions of conflict between a child and a parent (Simmons et al., 2019b). They found that the parents perceived any physical aggression, psychological coercion or intimidation, and financial abuse to be abusive behavior if they happened a few times a year, whereas verbal aggression had to occur daily. Simmons et al. (2019a) studied what Australian parents considered abusive YPA, and future studies should confirm whether abusive behavior in the YPA context varies across cultures.

### Child-to-Parent Violence Questionnaire (CPV-Q; Contreras et al., 2019)

This questionnaire consists of 14 parallel items (father/mother) with four factors: Psychological (4 items), Physical (3 items), and Financial Abuse (3 items), as well as the Control and Domain dimension (4 items) (e.g., “I have told my parents that at home, they have to do what I want”). Adolescents are asked to specify how often they have perpetrated each of the behaviors against their parents in the past year using a 4-point Likert-type scale (0 = *Never*, 1 = *Rarely - it has occurred once*, 2 = *Sometimes* - *2 or 3 times*, 3 = *Many times - 4 or 5 times*, 4 = *Very often - 6 times or more*). Some authors indicate that control and domain over a parent is a key aspect of YPA (Cottrell, 2001; Molla-Esparza and Aroca-Montolío, 2018). This scale was administrated to children of 12–18 years from a community population.

## Critical Aspects of Instruments

All YPA assessment instruments show evidence on a two-factor model (physical and psychological aggression) except CVS, which is unidimensional (Edenborough et al., 2011). Although the CTS (Straus, 1979) were originally administered as a measure for various forms of family violence, the CTS-2 has specific items of intimate partner violence. Even though the CTS were applied to measure verbal and physical aggression against parents, did not include a dimension such as financial abuse or emotional abuse (control or coercive behavior). This instrument takes into account the frequency of the behavior rather than its severity, but YPA-specific instruments (e.g., ABC-I) have developed to assess potential abusive behavior.

The scientific literature shows some problems associated with a lack of consensus about the definition of YPA and the operationalization of some types of aggressive behavior. For instance, in some instruments financial abuse has been assessed as a dimension on its own (Ibabe et al., 2014; Contreras et al., 2019), in other instruments as an element of a physical-financial abuse factor (Ghanizadeh and Jafari, 2010), as psychological abuse (Calvete et al., 2013a), or as coercive behavior (Simmons et al., 2019b). This issue can be complex if it takes into account that psychological and emotional forms of abuse facilitate to dominate and exercise control over another person (Tolman, 1992).

Also, it is surprising that the Emotional Violence subscale of the IVS (e.g., “I blackmail my father to get what I want”) (Ibabe et al., 2014), the Control and Domain subscale of the CPV-Q (e.g.,” I have told my parents that at home, they have to do what I want”) (Contreras et al., 2019), the Coercive Behavior subscale of the ABC-I (e.g., “I have threatened to hurt myself or others if my parents did not do what I wanted”) (Simmons et al., 2019b) measure similar constructs. Examples of emotional abuse indicated by Kennair and Mellor (2007) were making the parent think he or she was crazy or employing manipulative threats. Although psychological and emotional abuses are sometimes used synonymously, the difference between psychological abuse and emotional abuse involves controlling and manipulative behavior. The eleven YPA tools used by researchers across different ages (from 10 to 25 years) include preadolescents, adolescents, and young adults. Internal consistency of the CPAQ’s subscales is detailed, but it sometimes does not reach the desirable level (α ≥ 0.70) (Calvete et al., 2015a; Izaguirre and Calvete, 2017). A risk assessment tool for YPA (CPVR, Loinaz and Sousa, 2020) was found, which could be useful to detect the development of violence or for managing the cases depending on risk level. Some of the problems detected in the assessment instruments are related to problems of conceptualization or to a lack of consensus among researchers.

Table 4 shows evidence of the psychometric properties of the eleven instruments, and according to COSMIN guidelines only two instruments (Violent Behavior Questionnaire and Adolescents’ parent-directed aggression) fulfilled the criteria for category C and should therefore not be recommended for use. All other instruments were placed in category B, but three instruments (CPV-Q, CPAQ, ABC-I) stand out positively. They may still be recommended, but further validation is needed.

## Conclusion and Implications

In the last two decades, scientific interest in YPA has grown exponentially but the theoretical foundation is weak. One of the biggest challenges for YPA is a lack of internationally agreed upon terminology and definitions, which makes it difficult to compare different studies (Moulds et al., 2019). This paper has tried to contribute to the field of YPA by offering a systematic review of the extant literature, describing theoretical and empirical limitations in the conceptualization, and the measures used.

One purpose of this study was to analyze the terms, definitions and typologies used in YPA research. Although in total 46 different terms were found (Table 2), the most appropriate term for adolescents and young adults directing their aggressive behavior toward a parent is *youth-to-parent aggression*. However, as in early childhood aggression less harm is caused and the consequences are not as serious, the parental role in this developmental stage is different from that of young people, as are the legal consequences for children and parents. For these reasons, the proposed term for children under 12 who assault their parents is *child-to-parent aggression*. Aggression by young children hardly origins physical injury, although it may cause emotional distress to parents and continues in adolescence and adulthood as dating violence and intimate partner violence (Ulman and Straus, 2003). The word ‘abuse’ implicitly suggests a person who is an abuser (Holt, 2011). Thus, it may not be appropriate in some cases of YPA, especially when children are under 12 years of age. It is important to differentiate abusive and non-abusive YPA, taking frequency and severity into account. Some studies have measured the relative frequency and severity of YPA situations (Kolko et al., 1996; Gebo, 2007; Calvete et al., 2013a; Simmons et al., 2019a; Ibabe et al., 2020). The presence of physical YPA can be considered abusive, but the presence of a single or infrequent non-physical behavior is not abusive. Specific incidents of aggression are claimed to be part of normative youth behavior, although cases of a continuous pattern of abusive behavior in youth-to-parent relationships would be considered abusive YPA. In other studies, the prevalence rates of interpersonal violence (interparental violence, dating violence, and YPA) have been calculated using the zero tolerance criteria (using violence at any point in the last year) and technical abuse criteria (if the response “sometimes” or more in terms of frequency was stated in response to any item) (Ibabe, 2019; Ibabe et al., 2020). Similarly, Beckmann (2020) also used considered zero tolerance criteria (“once or twice”) and the technical abuse criteria (“three times” or more) to calculate YPA prevalence rates. Nevertheless, the youth-to-parent abuse term could be reserved for a diagnosis of abuse using an instrument with adequate psychometric properties as a function of country (Australia, ABC-I, Simmons et al., 2019a; Spain, CPAQ, Calvete et al., 2013a) or any technical abuse criterion. ABC-1 (Simmons et al., 2019a) includes a cut-off score to identify abuse, while CPAQ (Calvete et al., 2013a) considers severe physical aggression if physical aggression has happened at least three times in the previous year, and severe psychological aggression if it has happened at least six times in the same period.

After performing a systematic review of the existing definitions, thirteen definitions are analyzed to establish a full definition of YPA, distinguishing among abusive YPA and non-abusive YPA. Youth-to-parent aggression is defined as aggressive behavior (physical, psychological, emotional, financial, or sexual) by young people toward a parent or caregiver consciously and repeatedly over time, when parents and children usually live together. Youth-to-parent abuse is defined in the same way as YPA, but with young people perpetrating physical aggression or frequent non-physical aggression toward parents. Although to consider youth-to-parent abuse, it would be recommendable to make the diagnosis of abusive YPA using any instrument (CPAQ, Calvete et al., 2013a; ABC-I, Simmons et al., 2019b) or technical abuse criteria (Beckmann, 2020; Ibabe et al., 2020).

YPA and intimate partner violence occur in the context of interpersonal relationships, and they have conceptual similarities concerning the nature of violence (physical, psychological, emotional, economic, or sexual), typologies of YPA (Johnson, 2008), as well as empirical evidence on gender symmetry in intimate partner violence (Straus, 2010), or the profiles of perpetrators and victims of YPA and intimate partner violence. Sometimes financial aggression is considered as psychological aggression (e.g., Calvete et al., 2013a). Two unique features of YPA are the parent’s legal responsibility with respect to the child and the need to prioritize the needs of child in any intervention (Holt, 2013). The intentionality to harm the victim and repeated violent behavior are necessary to consider maltreatment (Molla-Esparza and Aroca-Montolío, 2018). Nevertheless, although YPA is not a deliberate and intentional strategy of children, if they use it as a way of persuading their parents to fulfill their wishes, parents could feel absolutely disorientated and disempowered.

This study has revealed that four typologies of YPA (Offensive, Defensive, Affective, and Situational) could be discriminated as a function of the coercion level and nature or directionality of the violence. Offensive YPA is similar to intimate terrorism (Johnson, 2008), with children exercising coercive control or emotional violence toward their parents and the parents living under constant threat. This typology has some similarities with intimate partner violence regarding power and control over the victim and is characterized by the manipulation of other persons for own advantage. Moreover, it is characterized by proactive aggression, designated as instrumental, deliberated, and scheduled (Ramírez and Andreu, 2006). In any case, the two are deliberate actions directed at reaching a specific goal. Intervention programs should support the involved families to empower the parents and enable them to control their children’s behavior. Defensive YPA includes violent behavior for self-defense in child abuse experiences or to defend another person in interparental violence situations. There is considerable empirical data about the association between YPA and physical punishment (Calvete et al., 2015b; Ibabe, 2019) or interparental violence exposure (Boxer et al., 2009; Ibabe and Jaureguizar, 2011; Gallego et al., 2019; Ibabe et al., 2020). It is necessary to intervene with the parents to reduce neglectful practices. Affective YPA is characterized by children with problems controlling themselves, but the parents do not use violence to defend themselves. In these cases, it would be recommendable that interventions focus on anger controlling and social skills deficits in young people (Brown and Parsons, 1998), as well as training parents in positive communication strategies (Calvete and Orue, 2016). Situational YPA is a minor form of bidirectional violence without abusive behavior by parents or children, where parents and children are unskilled in arguing, listening to each other, and not sufficiently socially skilled. Families experiencing situational violence could obtain help from early intervention for YPA situations (Ibabe et al., 2018), learning effective conflict-resolution strategies and communication skills carried out by trained mental health professionals. In the Trait-Based Model (Kuay et al., 2017), the perpetrators of YPA are separated into “generalists” (with high on callous-unemotional features, perpetrate YPA as well as violence outside the family), and “specialists” (with low on callous-unemotional features and specifically YPA). Moulds et al. (2019) found that the majority of YPA offenders are antisocial (e.g., they have other offenses), while that YPA crime in isolation is infrequent.

When YPA is conceptualized as violent incidents without exploring their context in community population and their frequency or severity (e.g., “How often in the past year have you slapped a parent?”), gender symmetry between male and female perpetrators has been reported in numerous studies (Ulman and Straus, 2003; Ibabe and Jaureguizar, 2011). Nevertheless, when cases of YPA involve a greater occurrence and severity of aggression, parents reach a “breaking point” and seek help through the police or other services (Holt, 2013; Howarth and Feder, 2013), and such cases involve sons more often than daughters (Walsh and Krienert, 2007; Ibabe and Jaureguizar, 2010; Condry and Miles, 2014). In the last decade, some agencies traditionally concerned with intimate partner violence or domestic violence have been aiding women abused by their children (Gallagher, 2008). Therefore, the term ‘parent’ hides the reality that it is most frequently ‘mother’ who is the victim of such abuse (Holt, 2011). As almost all assessment instruments of YPA have parallel items directed toward father versus mother, it would be interesting to provide data on both youth-to-father aggression and youth-to-mother aggression. It is necessary to point out that sometimes children direct their violent behavior toward both parents, siblings or grand-parents (Ibabe and Jaureguizar, 2010).

Other objectives were to show the psychometric properties of instruments identified in the systematic review to assess YPA, and to identify the best instruments using the COSMIN protocol. Table 4 shows the eleven instruments found with any information about their psychometric properties. Among these YPA assessment tools, three were identified as the most promising instruments (B category, can be administrated with caution) to be used in the research or clinical context: *Child-to-parent Violence Questionnaire* (CPV-Q, Contreras et al., 2019), *Adolescent Child-to-Parent Aggression Questionnaire* (CPAQ, Calvete et al., 2013a) and *Abusive Behavior by Children-Indices* (ABC-I, Simmons et al., 2019a). In general, it is necessary to conduct more cross-cultural studies, but it would be important to unify the conceptualization of YPA and the age limit. In this context, psycho-emotional aggression could include different types of behavior as ignoring parents, rejection, or non-verbal expressions of contempt (Aroca-Montolío et al., 2014).

YPA is a complex social problem, which currently involves many controversies. For example, criminology presents teenagers as potential delinquents in the public context, but not within the home (Condry and Miles, 2014). The subject of YPA might be a cultural taboo (Edenborough et al., 2008) because it is seen by some people as “unnatural and almost inconceivable,” taking into account the supposed authority of parents (Pagani et al., 2004). However, the fact of not understanding a phenomenon like YPA does not mean that it does not exist. Parent victims of YPA are legally obliged to live together with their child offender until they reach the age of majority (Coogan, 2011), a fact that increases parental vulnerability. This vulnerability will be higher when there are children with serious mental illness or with drug abuse. The importance of parental misconduct (from dysfunctional parenting to child abuse) as a causal factor in YPA and parricide has not been central in the academic discourse (Holt and Shon, 2016). Nevertheless, a broader perspective regarding the sources of family conflict is necessary to advance YPA research. If the child-parent conflicts are not managed satisfactorily in adolescence, they will continue in early adulthood, adulthood, and old age.

As a systematic review provides an unbiased assessment of the studies across countries, this is a relevant strength of current study. This type of research can add knowledge to the scientific community especially when there are gaps in the existing conceptualization. However, the community’s response to YPA is different depending on the country, which could thus represent a limitation of the current analysis. In general, the conceptualization of YPA could be different in juvenile justice, child welfare or domestic violence contexts (Hunter et al., 2010) or at least in the social representation of mother as victims and children as perpetrators in different services providers. In any case, this could be an interesting goal for a further study.

In conclusion, it is essential to establish a broad consensus on the definition and measurement of YPA to improve researchers’ capacity to effectively build on existing evidence (O’Hara et al., 2017). This implies improving the conceptualization of YPA and measuring this type of abuse consistently, as well as avoiding the arbitrary age-related boundaries of YPA perpetration. Previous literature reviews (Kennair and Mellor, 2007; Hong et al., 2012; Simmons et al., 2018) conclude that results across studies using different definitions of YPA have led to contradictory findings. Operational variables used in the studies do not reflect a theoretical construct. The four typologies of YPA which have been proposed (Offensive, Defensive, Affective, and Situational) can help in the initial psychodiagnosis of a YPA situation and prevent more severe situations of YPA requiring early intervention. There is empirical evidence that supports YPA as the intermediary stage in the intergenerational transmission of violence (Gebo, 2007; Ibabe et al., 2020). Moreover, in a few cases parricide may be the final-stage culminating action for children (Walsh and Krienert, 2009). It is therefore necessary for practitioners, parents, and children alike to identify and name YPA to break the silence concerning this hidden family abuse. It needs to be expressed sincerely in a safe context with joint goals of enhancing communication and building respectful interactions. In our society, children are seen as potential victims and the parents have supremacy of power (Tew and Nixon, 2010). Thus, it could be difficult to understand how parents may become afraid of their own children, but keeping in mind the YPA typologies would help in that understanding of some situations. Future research should integrate the research of aggression in other contexts and investigate what is distinctive to YPA, as well as confirm whether the profile of the perpetrator of intimate partner violence is analogous to the profile of YPA perpetrator. Furthermore, it would be interesting to analyze the extent to which YPA is bidirectional or unidirectional, and the prevalence rate should also be taken into account in this point of view.

## Author Contributions

The author confirms being the sole contributor of this work and has approved it for publication.

## Conflict of Interest

The author declares that the research was conducted in the absence of any commercial or financial relationships that could be construed as a potential conflict of interest.
